# Microbial diversity and community composition of fecal microbiota in dual-purpose and egg type ducks

**DOI:** 10.3389/fmicb.2023.1092100

**Published:** 2023-03-31

**Authors:** Jing Ouyang, Yuhang Li, Yongfei Wu, Hongbo Tang, Sumei Zheng, Yanpeng Xiong, Luping Wang, Cong Wang, Keyi Luo, Yuren Gao, Xueming Yan, Hao Chen

**Affiliations:** Jiangxi Key Laboratory of Bioprocess Engineering, College of Life Sciences, Jiangxi Science and Technology Normal University, Nanchang, China

**Keywords:** egg type duck, dual-purpose type duck, gut microbiota, amplicon sequencing, functional prediction

## Abstract

**Introduction:**

Ducks are important agricultural animals, which can be divided into egg and dual-purpose type ducks according to economic use. The gut microbiota of ducks plays an important role in their metabolism, immune regulation, and health maintenance.

**Methods:**

Here, we use 16S rDNA V4 hypervariable amplicon sequencing to investigate the compositions and community structures of fecal microbiota between egg (five breeds, 96 individuals) and dual-purpose type ducks (four breeds, 73 individuals) that were reared under the same conditions.

**Results:**

The alpha diversity of fecal microflora in egg type ducks was significantly higher than that in dual-type ducks. In contrast, there is no significant difference in the fecal microbial community richness between the two groups. MetaStat analysis showed that the abundance of Peptostreptococcaceae, Streptococcaceae, *Lactobacillus, Romboutsia*, and *Campylobacter* were significantly different between the two groups. The biomarkers associated with the egg and dual-purpose type ducks were identified using LEfSe analysis and IndVal index. Function prediction of the gut microbiota indicated significant differences between the two groups. The functions of environmental information processing, carbohydrate metabolism, lipid metabolism, xenobiotic biodegradation and metabolism, and metabolism of terpenoids and polyketides were more abundant in egg type ducks. Conversely, the genetic information processing, nucleotide metabolism, biosynthesis of amino acids and secondary metabolites, glycan biosynthesis and metabolism, fatty acid elongation, and insulin resistance were significantly enriched in dual-purpose type ducks.

**Discussion:**

This study explored the structure and diversity of the gut microbiota of ducks from different economic-use groups, and provides a reference for improving duck performance by using related probiotics in production.

## 1. Introduction

Poultry genetic resources are an important animal genetic resource. Ducks, chickens, and geese have become the major poultry consumed in China. It is believed that domestic ducks were domesticated from *Anas platyrhynchos* and *Anas Poecilorhyncha* (Hackett et al., 2008). There are 32 indigenous duck breeds in China, accounting for half of the world’s duck breeds. Domestic ducks play an essential role in providing people with eggs, meat, and duck down (Huang et al., 2013). Duck is the second largest poultry product after chicken, and duck breeding has gradually become an important component of animal husbandry in China.

Many local domestic duck populations exhibit differences in body size, plumage color, and economic use due to the diversity in geography, ecological conditions, and various directional selections in China. Chinese ducks can be divided according to economic use into egg type (mainly used in the production of duck eggs) and dual-purpose type (used in the production of duck meat and eggs) ducks, with unique gut microbiota which may be one of the key reasons enabling them to stay healthy and adapt to various environments. Previous studies have shown that microbial cells living in the gastrointestinal tract (GIT), known as the microbiota, exhibit important functions in host adaptation (Xin et al., 2019). The gut microbiota can absorb and convert the indigestible dietary polysaccharides into short-chain fatty acids (SCFAs), which can be utilized as energy and carbon sources by the host (Hooper et al., 2002). Therefore, the gut microbiota can promote nutrient metabolism, maintain the gastrointestinal mucosal barrier, and enhance the immune response of domestic ducks (Cerf-Bensussan and Gaboriau-Routhiau, 2010). Various factors influence the colonization and stability of the gut microbiota of domestic ducks, including age, nutrition, antibiotics, probiotics, and the management of poultry houses (Raza et al., 2019; Zhu et al., 2020). Additionally, the gut microbiota is closely related to the growth and development indices of poultry. The abundances of *Escherichia*/*Shigella* were found to correlate negatively with growth and fat digestibility in broiler chickens (Rubio et al., 2015). Moreover, the colonization of *Campylobacter* in broiler chickens has been associated with reduced economic performance in terms of an increase in cumulative feed conversion ratio (FCR) (Awad et al., 2015). However, the relationship between the composition and diversity of gut microbiota and production performance of poultry is unclear (Diaz Carrasco et al., 2019). Therefore, determining the community structure and diversity of the gut microbiota of Chinese ducks may provide key insights into their health, development, and production performance.

The development of high-throughput sequencing (HTS) has enabled researchers to gain an understanding of the compositional and metabolic characteristics of the gut microbiome, which leads to a better understanding of the interactions between the gut microbiome, environment, and host. In a study on the GIT microbiome of ducks, significant changes in microbial communities and SCFAs between different GITs were shown to potentially be related to differences in gut function (Yang et al., 2020). Additionally, Zhu et al. revealed that the gut microbiome characteristics of Gaoyou ducks at different developmental stages and segments of the small intestine were significantly different (Zhu et al., 2020). Current studies of the gut microbiota of ducks predominantly focus on a few breeds of ducks, investigating the effects of different growth periods, intestinal segments, feeding conditions, and diet on the intestinal microbiota (Allen et al., 2010; Qin et al., 2020). However, there are few studies focusing on the gut microbiota of ducks from different economic-use groups.

In this study, we investigate the composition and diversity of the fecal microbiota from 169 indigenous ducks (9 breeds) divided into egg type and dual-purpose type ducks,using 16S rDNA sequencing based on the Illumina Novaseq 6,000 sequencing platform. The microbial functions were predicted based on 16S rRNA gene composition and abundance. The purpose of this study was to explore the differences in gut microbiota between egg and dual-purpose type ducks and provide a reference for the protection and utilization of indigenous duck breeds.

## 2. Methods and materials

### 2.1. Experimental design and sample collection

**Figure 1:**
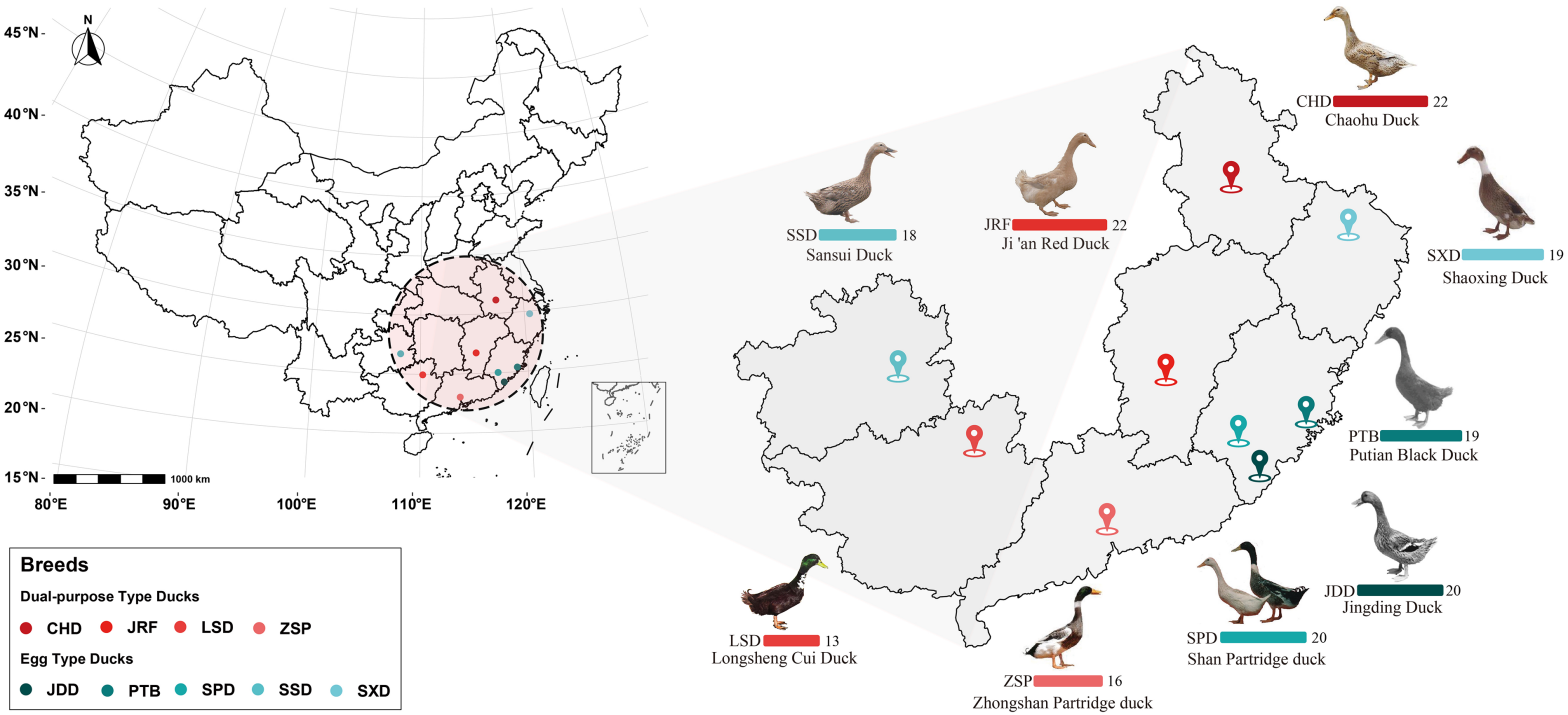
Source area overview of the 169 gut microbiome samples from the nine Chinese duck breeds. Bar charts represent the number of fecal samples from each duck breed from the different provinces colored according to the legend.

This study conformed to the guidelines for the care and use of experimental animals established by the Ministry of Science and Technology of the People’s Republic of China (approval number: 2006–398). The research protocol was reviewed and approved by the ethical committee of Jiangxi Science and Technology Normal University. A total of 169 fecal samples from nine Chinese breeds were collected from the National Waterfowl Resource Bank in Quanzhou, Fujian Province in October (Han et al., 2018). The samples were divided into egg type and dual-purpose type ducks according to the description of economic use in *Animal Genetic Resources in China: Poultry*. The source area and sample information are presented in Figure 1 and Supplementary Table S1. To minimize potential variation in microbiota composition due to feeding and other environmental factors, all the ducks in this experiment were raised on the same farm and fed with the same batch of feed. In addition, the domestic ducks of the same breed lived in the same duck house. All the flocks were healthy based on regular veterinary inspections during the study and none had been treated with antibiotics on the layer farm. We pressed the abdomen and rectum of duck to stimulate excretion and collected 2–5 g fresh fecal contents with non-liquid state using sterile collection tubes on the 80th day after hatching. Once a sufficient amounts of feces samples were collected, they were immediately stored in liquid nitrogen. After sampling, all samples were stored in the −80°C refrigerator until DNA extraction.

### 2.2. DNA extraction, 16S rDNA gene amplification, and NovaSeq sequencing

Total genomic DNA of the gut microbiota from 169 Chinese ducks was extracted using the CTAB/SDS method. The DNA concentration and purity was evaluated on 1% agarose gels and using a NanoDrop 2000 (Thermo Scientific, United States). The genomic DNA was diluted to 1 ng/μL using sterile water before amplification of the 16S rDNA.

The hypervariable region (V4) within the 16S rRNA gene was amplified using universal eubacterial primers. The forward primer 515F (5′-GTGCCAGCMGCCGCGGTAA-3′) and reverse primer 806R (5′-GGACTACHVGGGTWTCTAAT-3′) were used with additional overhang adapters and unique 10 nt barcodes to allow multiple samples to be analyzed on a single platform. PCR reactions comprised of 3 μL of the forward and reverse primers (2 μM), 10 μL genome DNA (1 ng/μL), and 15 μL 2 × Phusion^®^ High-Fidelity PCR Master Mix (New England Biolabs, United States). The thermocycling conditions consisted of an initial denaturation at 98°C for 1 min; followed by 30 cycles of 98°C for 10 s, 50°C for 30 s, and 72°C for 30 s; and a final extension at 72°C for 5 min. Then, PCR products mixed in equidensity ratios were purified using a Qiagen Gel Extraction Kit (Qiagen, Germany). The amplicons were processed for sequencing library preparation using TruSeq^®^ DNA PCR-Free Sample Preparation Kit (Illumina, United States) and the index codes were added. The quality of the sequencing libraries was assessed using a Qubit 2.0 Fluorometer (Thermo Scientific, United States) and Agilent Bioanalyzer 2,100 system. The library sequencing was performed using an Illumina NovaSeq platform and 250 bp paired-end reads were generated.

### 2.3. Sequence data processing

Dirty data is present in raw paired-end data obtained by Illumina NovaSeq sequencing. To make the results more accurate and reliable, the raw amplicon reads were assigned to their own samples using their unique barcode, then the barcode and primer sequences were removed from the 5′ and 3′ ends. The paired-end reads were merged using FLASH (V1.2.7) (Magoc and Salzberg, 2011) and the spliced sequences were called raw tags. The high-quality clean tags were obtained using the QIIME (V1.9.1) (Caporaso et al., 2010). The tags were compared with the reference database (Silva database, https://www.arb-silva.de/) using the UCHIME algorithm(Edgar et al., 2011) to detect and remove the chimera sequences. After this process, the effective tags were obtained.

The Uparse software (V7.0.1001) (Edgar, 2013) was used to cluster preprocessed effective tags into operational taxonomic units (OTUs) exhibiting 97% similarity. Representative sequences for each OTU were screened using the Silva Database (Quast et al., 2013) based on Mothur algorithm to further annotate taxonomic information. OTU abundance information was normalized using a standard of sequence number corresponding to the sample with the least sequences. Subsequent analysis of alpha and beta diversities was performed on this normalized data.

### 2.4. Statistical analysis

In order to understand the characteristics of fecal microbial community structure in ducks, a normalized OTUs representative table was analyzed. We identified species with specific or shared OTUs between different samples types using venn diagram generated by VennDiagram package (Chen, 2018). Moreover, alpha diversity was applied in analyzing the complexity of species diversity for each sample. Then, Principal coordinate analysis (PCoA) (Minchin, 1987) based on Weighted Unifrac dissimilarities at the OTU-level was performed to evaluate the differences between samples with regards to species complexity. Significant changes in community structure were evaluated using Wilcox rank sum test (Hothorn et al., 2017), multi response permutation procedure (MRPP) (O'Reilly and Mielke, 1980), analysis of similarities (ANOSIM) (Chapman and Underwood, 1999), and permutational MANOVA (ADONIS) (Stat et al., 2013).

In addition, we identified economic use specificity bacteria. Firstly, we performed t-test (equal variance) to identify different bacteria phyla of top 10 relative abundance, and used Benjamini Hochberg FDR to correct *p* value. To further evaluate the uniqueness at the bacterial family level for a given sample group, their indicator value (IndVal) index was determined, which took into account the abundance of a taxon in a community and frequency of occurrence in all communities (Chen et al., 2020). The IndVal index measured the particularity of taxa from those found in only a single community to those found across all communities and was calculated using the indicspecies package (Cáceres and Legendre, 2009), to evaluate the differences of microbiota from the two economic-use populations. The Welch’s t-test was performed to determine the significant differences of gut microbiota at dominated family and genus using the statistical analysis of metagenomic profiles (STAMP) (Parks et al., 2014) software, and value of p was corrected by Benjamini Hochberg FDR. We determined the core microbiota of ducks in this study, the bacterial genus abundance >0.1 and > 1.0% were taken into account, and the number of different core microbiota was calculated by Welch’s t-test. To identify different biomarkers for different duck breeds and economic uses, the bacterial abundance profile of ducks pooled by (i) breed and (ii) economic use were analyzed using linear discriminant analysis effect size (LEfSe) (Segata et al., 2011). We made sure the non-parametric factorial Kruskal-Wallis (KW) sum-rank test and Wilcoxon rank sum test’s *p* value <0.05, and LDA Score > 3.5.

Finally, we evaluated the functional characteristics of duck fecal microbe. The annotated results of the amplifier were associated with the corresponding functional database, and functional prediction of the microbial community in the samples was performed using Tax4Fun (Aßhauer et al., 2015), a R package for functional prediction of environmental samples such as intestinal and soil samples, based on the 16S Silva database. The Welch’s t-test was used to analyze the differences of the Kyoto Encyclopedia of Genes and Genomes (KEGG) secondary metabolic pathways between egg and dual-purpose type ducks using STAMP software, and used FDR to correct *p* value.

## 3. Results

### 3.1. Quality and classification of sequenced samples

The 169 fecal extracts were sequenced using a paired-end approach on Illumina Novaseq sequencing platform (Figure 1). An average of 70,711 tags were obtained from each sample through read splicing. A total of 70 387 clean tags were obtained after quality control, with a quantity of 63 162 effective tags and a quality control efficiency of 88.42% (Supplementary Table S1). A total of 13 460 OTUs clustered with 97% similarity and were annotated using the Silva138 database. Among the annotation results, 80.55% were annotated to phylum level, 58.47% to family level, and 37.60% to genus level (Table 1).

**Table 1:** Summary statistics of annotated species.

Annotation lists	Total
OTU catalogue	13 460
Annotated on database	12 706 (94.40%)
Annotated on unclassified	754 (5.60%)
Annotated on kingdom level	94.40%
Annotated on phylum level	80.55%
Annotated on class level	78.30%
Annotated on order level	71.02%
Annotated on family level	58.47%
Annotated on genus level	37.60%
Annotated on species level	6.96%

The OTUs clustered at > 97% similarity were counted and annotated into Silva138 database using QIIME.

**Figure 2:**
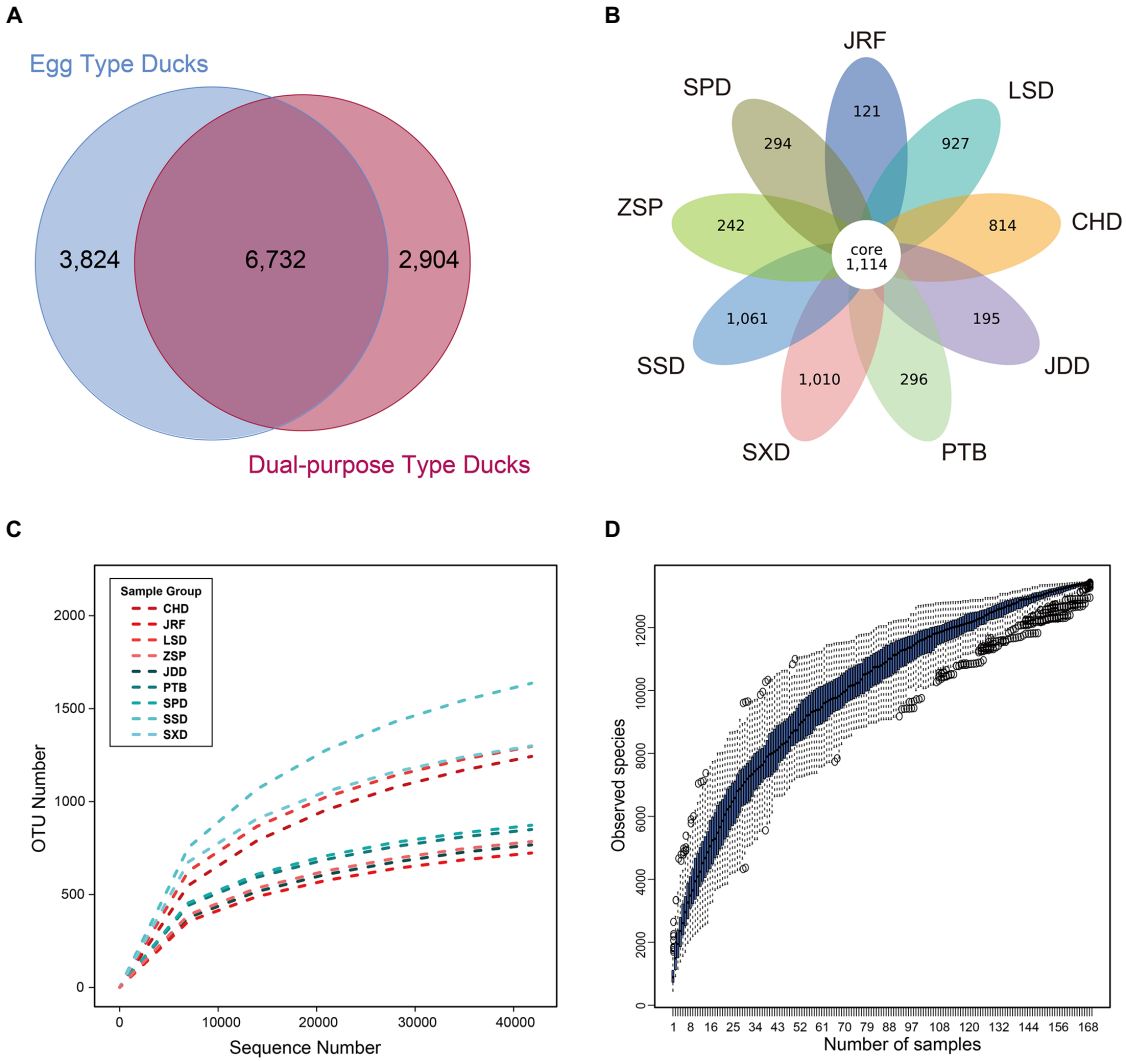
OTU classification and species richness analysis of the nine Chinese duck breeds. The Venn graph was drawn after homogenization processing for all samples. **(A)** Venn analysis of OTUs from dual-purpose and egg type ducks. **(B)** Venn analysis of OTUs from nine duck breeds. Each circle in the figure represents a group or breed, the numbers in circle and circle overlap represents the number of shared or unique OTUs in different samples. **(C)** Species rarefaction curves of the duck breeds. **(D)** Species accumulation curves of the current study.

The nine duck breeds were divided into egg type and dual-purpose type ducks. A total of 6 732 OTUs were common to both groups; 3 824 and 2 904 OTUs were unique to egg type and dual-purpose type ducks, respectively (Figure 2A). Additionally, the nine duck breeds shared a total of 1 114 core OTUs. The number of unique OUTs observed in Sansui Duck (SSD), Shaoxing Duck (SXD), Longsheng Cui Duck (LSD), and Chaohu Duck (CHD) was more than 800. The other five breeds displayed less than 300 unique OTUs (Figure 2B). The rank abundance curves of samples, breeds, and groups indicated high abundance and uniform distribution of species (Figure 2C and Supplementary Figure S1). The species accumulation curve was horizontal when samples reached 169, showing that the sample size selected in this experiment was sufficient (Figure 2D).

### 3.2. Richness and diversity of the fecal microbiome

**Figure 3:**
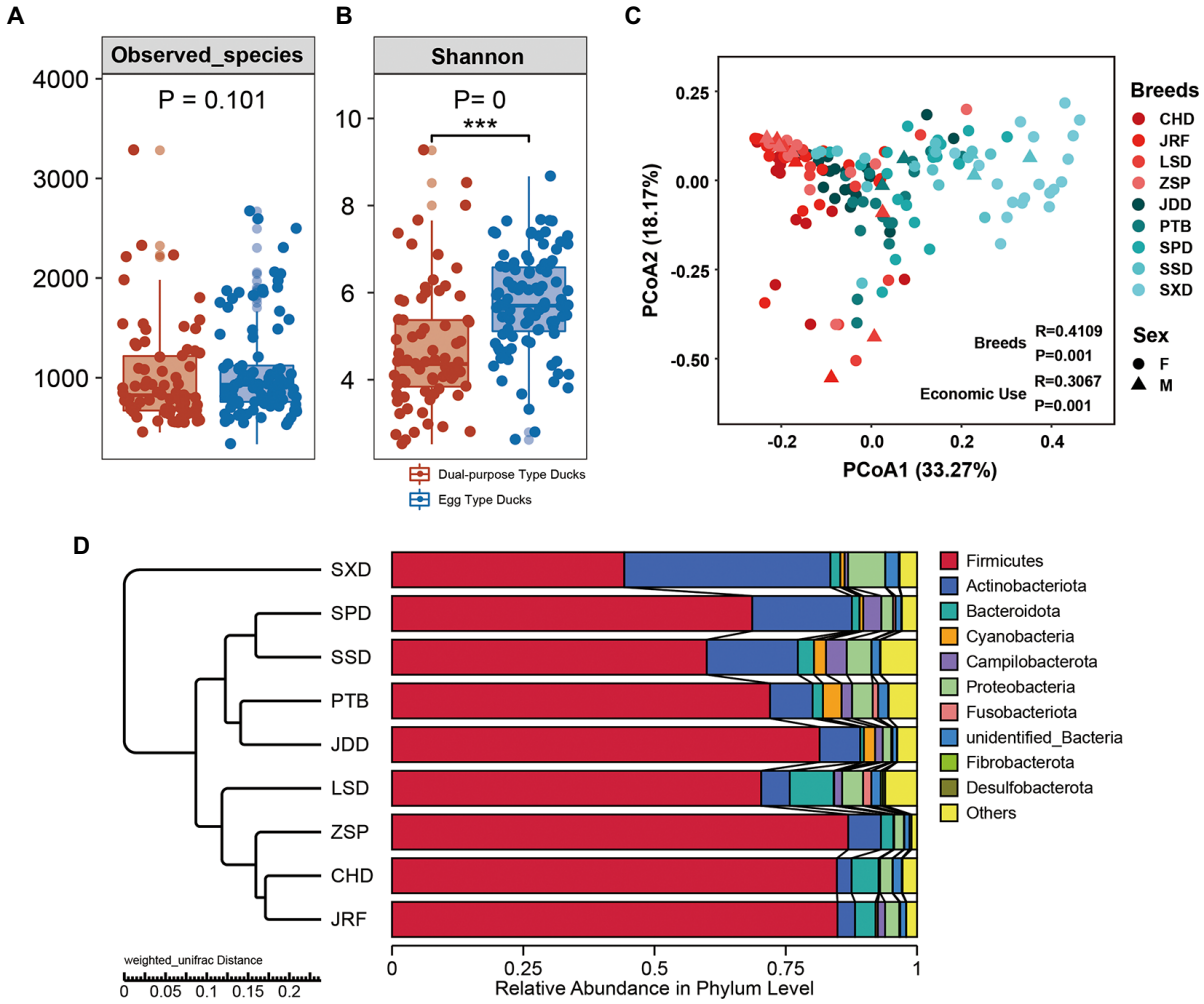
Alpha diversity and microbial community structures across dual-purpose and egg type ducks. **(A)** The observed richness and **(B)** Shannon diversity index of microbial communities in samples from domestic ducks. Principal Co-ordinates Analysis (PCoA) on Weighted Unifrac distances among sample types was plotted based on OTU abundances in **(C)** domestic duck samples. **(D)** Microbial community bar plot with cluster tree of the nine Chinese duck breeds.

The diversity of the gut microbiota of ducks was explored by evaluating different alpha diversity indicators at the OTU level (Figures 3A,B; Supplementary Figure S2A; Supplementary Table S2). Observed species index, representing bacterial community richness, showed that OTU richness of fecal microbiota in egg type and dual-purpose type ducks exhibited no significant difference (*p* = 0.101) (Figure 3A). However, OTU richness of fecal microbiota was significantly different between some breeds. In dual-purpose type ducks, the OTU richness of CHD and LSD was higher than that of Ji’an Red Duck (JRF) and Zhongshan Partridge duck (ZSP). In egg type ducks, the OTU richness of SSD and SXD was higher than that of Putian Black Duck (PTB), Shan Partridge duck (SPD) and Jingding Duck (JDD). CHD, LSD, SSD, and SXD displayed higher OTU richness than JRF, ZSP, PTB, SPD, and JDD (Supplementary Figure S2B). The Shannon index revealed that the diversity of the fecal microbiota in egg type ducks was significantly higher than that in dual-purpose type ducks (*p* < 0.001) (Figure 3B). The four dual-purpose type-duck breeds displayed lower bacterial diversity compared with that of the five egg duck-type breeds. LSD and SXD displayed the highest bacterial diversity in dual-purpose and egg type ducks, respectively (Supplementary Figure S2C).

The gut microbial community structure of the egg and dual-purpose type ducks were explored by calculating and visualizing the Weighted Unifrac distance based on the OTU level. PCoA and UPGMA cluster analysis showed significant changes in microbial structure between the egg and dual-purpose type ducks (Figures 3C,D; Table 2). Additionally, microbial community between Chinese indigenous duck breeds had significant distinctions (Figure 3C).

**Table 2:** Differences significance test of community structure between dual-purpose and egg type ducks.

Method	Results of significance test					
Anosim	***R*-value**	***p*-value**	**–**	**–**	**–**	**–**
	0.3067	0.001	**–**	**–**	**–**	**–**
MRPP	**A**	**observed-delta**	**Expected-delta**	**Significance**	**–**	**–**
	0.07714	0.6067	0.6574	0.001	**–**	**–**
Adonis	**Df**	**Sums Of Sqs**	**Mean Sqs**	**F.Model**	***R*** ^**2**^	**Pr(>F)**
	1 (168)	2.942 (13.791)	2.94203 (0.08209)	35.838	0.17582 (0.82418)	0.001

### 3.3. Microbial community composition

**Figure 4:**
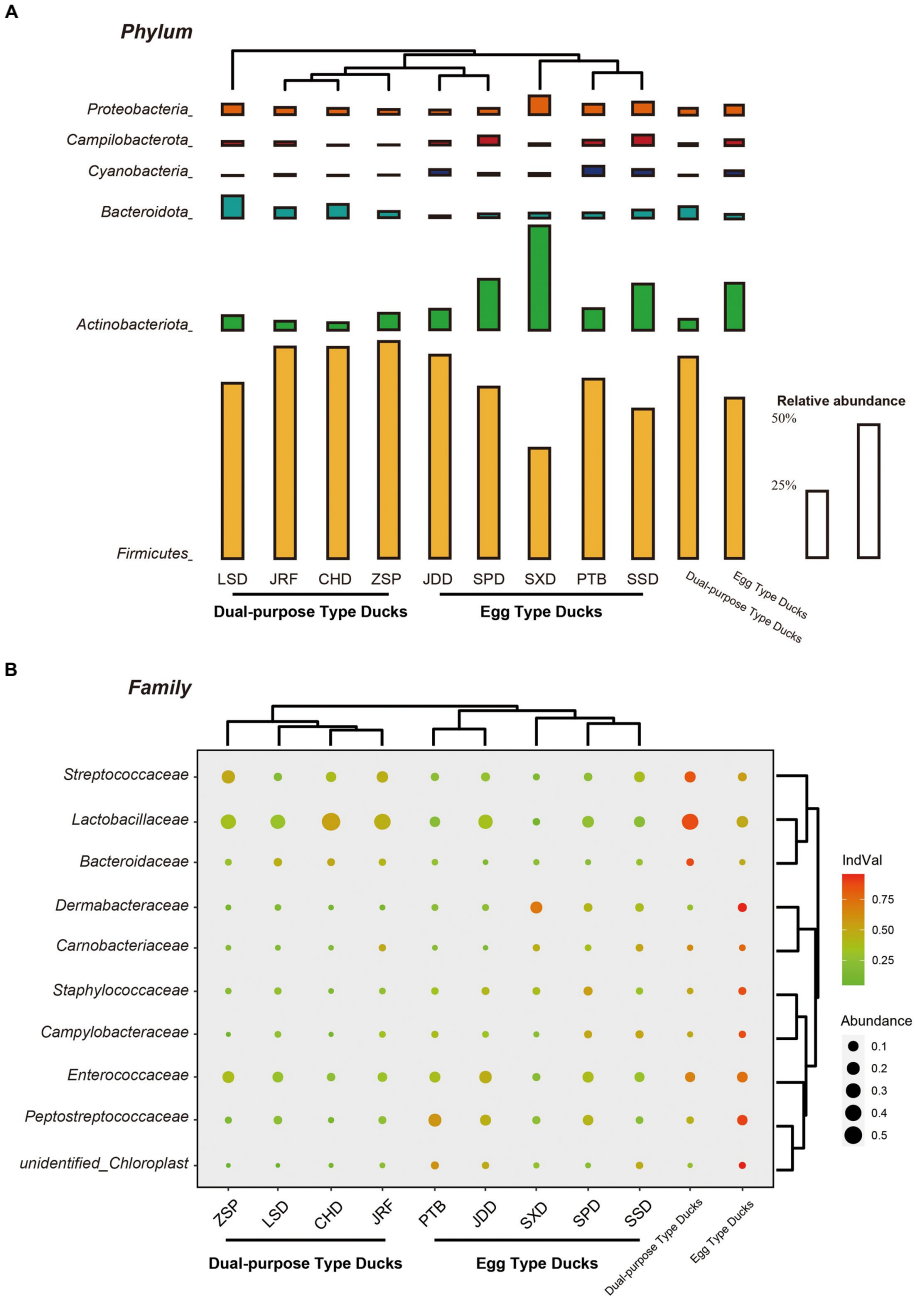
Microbial compositions of Chinese ducks. The average relative abundances of the most prevalent bacterial **(A)** phyla (bar length) and **(B)** families (circle size) in each sample type (indicated as above bubble plot) plotted for samples from the two groups and nine breeds. Cluster tree of domestic ducks showed similarities in the structure and composition of the fecal microbiota. The indicator value index (shading of circle color) represents the strength of association between a taxon and a given sample type, with larger values indicating greater specificity.

The change in gut microbial composition between two populations was evaluated by comparing the top 10 bacterial phyla, families, and genera with the highest abundances. At the phylum level, Firmicutes, Actinobacteria, Bacteroidetes, and Proteobacteria were found to dominate the microbial communities, accounting for more than 80% of the total microbial community (Figure 4A). Additionally, the microbial communities of the nine duck breeds clustered based on their economic-use groups. Statistical analysis showed that the proportion of Firmicutes in the dual-purpose type ducks was significantly higher than that in the egg type ducks (*p* < 0.001), while the proportion of Actinobacteriota in the dual-purpose type ducks was significantly lower than that in the egg type ducks (*p* < 0.001) (Supplementary Figure S3). The relative abundance of Fusobacteriota, Desulfobacterota, Fibrobacterota, and Bacteroidota in LSD was higher than that of other breeds. The relative abundance of Firmicutes was the lowest in SXD, but this breed exhibited the highest Actinobacteriota relative abundance (data not shown).

Lactobacillaceae, Peptostreptococcaceae, Streptococcaceae, and Enterococcaceae were revealed to be the most dominant bacterial groups at the family level in the fecal samples. The specificity of a taxon to a given sample type was measured by determining its IndVal index. The IndVal index and MetaStat analysis consistently showed that the IndVal index of Lactobacillaceae (IndVal = 0.872) and Streptococcaceae (Indval = 0.838) in dual-purpose type ducks was significantly higher than that of egg type ducks, while Peptostreptococcaceae (Indval = 0.894) was the opposite. Additionally, Bacteroidaceae (Indval = 0.876) were enriched in the intestinal tract of dual-purpose type ducks, while the abundance of Dermabacteraceae (Indval = 0.955), Carnobacteriaceae (Indval = 0.775), Staphylococcaceae (Indval = 0.867), and Campylobacteraceae (Indval = 0.871) was higher in the egg type ducks (Figure 4B; Supplementary Table S3).

**Figure 5:**
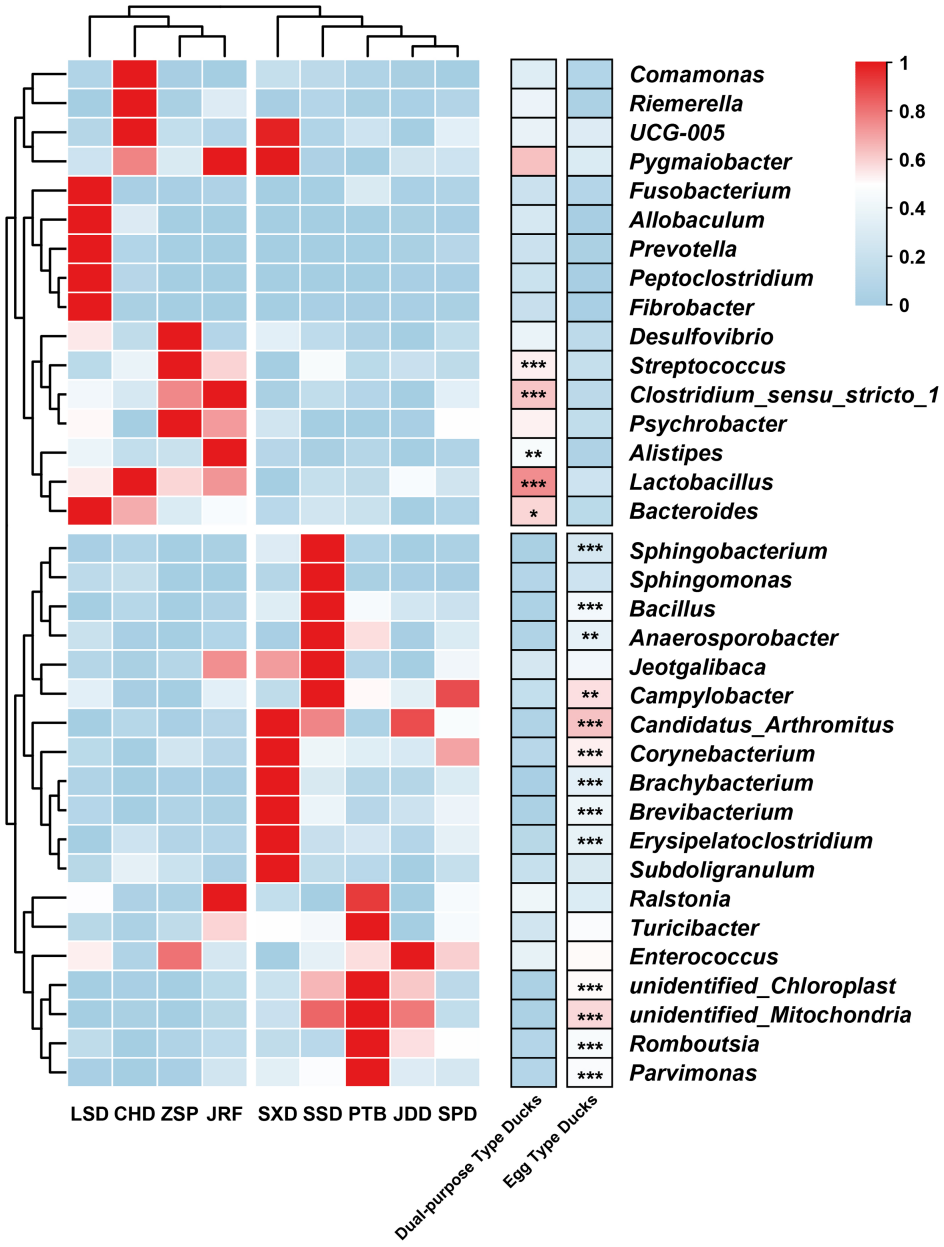
Heatmap of the 35 most abundant microbial genera. The genera with significant differences were determined using permuted t-statistics, and value of *p* was corrected by Benjamini and Hochberg FDR (^∗^ *p* < 0.05; ^∗∗^ *p* < 0.01; ^∗∗∗^ *p* < 0.001 for permuted t-statistics).

*Lactobacillus*, *Romboutsia*, *Streptococcus,* and *Enterococcus* were the most common genera (Supplementary Table S4). The proportion of *Lactobacillus* and *Streptococcus* in dual-purpose type ducks were significantly higher than that in egg type ducks, while *Romboutsia* was the opposite. There were 18 genera with significant differences among the 35 most abundant microbial genera (Figure 5). Consistent with the results at the family level, different the duck breeds clustered according to their economic use-group.

### 3.4. The core fecal microbiome and specific biomarkers

In total, 39 genera represented more than 0.1% abundance in the core fecal microbiome of two groups, combined with samples from all breeds. Twenty-three genera belonged to Firmicutes, representing 16 different families, eight genera to the Actinobacteriota, four genera to Proteobacteria, and the remaining four genera belonged to other diverse phyla. Additionally, 26 genera exhibited significant differences between egg and dual-purpose type ducks (Supplementary Table S4). Only five genera were detected within the core fecal microbiome between the two groups when the threshold was increased to 1% abundance. Four genera belonged to the phylum Firmicutes, one genus belonged to Actinobacteriota, and only Enterococcus exhibited no significant differences between the egg and dual-purpose type ducks.

**Figure 6:**
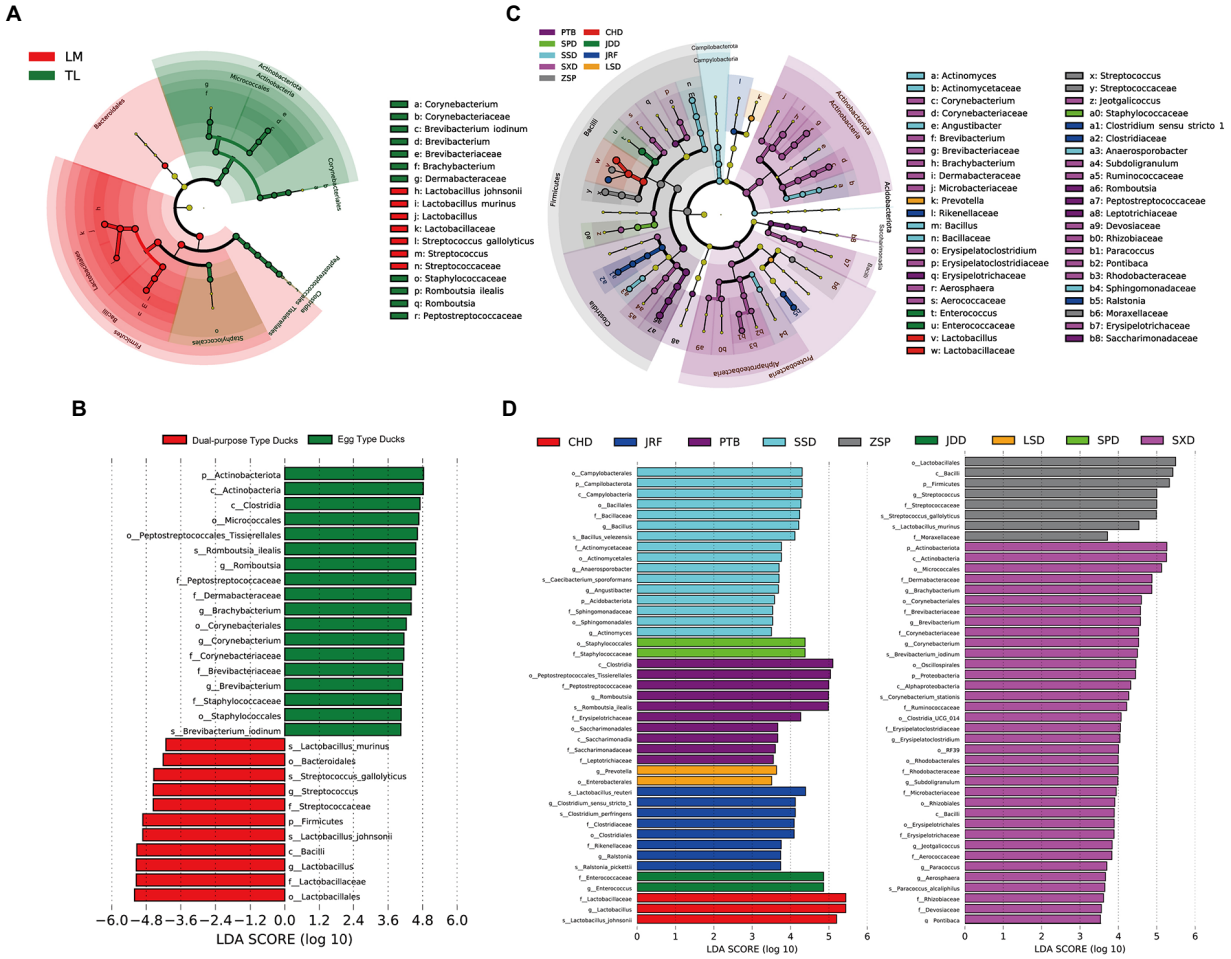
The biomarkers in Chinese ducks’ gut microbiota determined by LEfSe among two groups and nine breeds. The gut microbiota of **(A)** two groups and **(C)** nine breeds were compared. Colors indicate taxa or branches of the tree, which represent more significant groups. The size of each node represents their relative abundance. The gut microbiota of two groups **(B)** and nine breeds **(D)** were compared and determined as biomarkers using Kruskal-Wallis test (*p* < 0.05) with LDA score > 4 (**B**) and 3.5 (**D**).

LEfSe analysis was performed to identify specific taxa that varied in abundance consistently across the different duck breeds, and as a result could be used as biomarkers. In total, 29 specific biomarkers were identified in the two groups with LDA scores >3.5 (Figures 6A,B; Supplementary Figure S4). There were 18 and 11 biomarkers in the egg and dual-purpose type ducks, respectively. The genera *Corynebacterium*, *Brevibacterium*, *Brachybacterium,* and *Staphylococcaceae* were biomarkers in the egg type ducks, with *Streptococcus* as a biomarker in the dual-purpose type ducks. Additionally, the *Romboutsia* was a valuable intestinal biomarker in egg type ducks, while *Lactobacillus* was determined as a biomarker in dual-purpose type ducks. Moreover, the LDA threshold was set as 3.5 to detect specific biomarkers of Chinese duck breeds. A total of 87 specific biomarkers were identified, among which the maximum number of 36 biomarkers was identified in SXD; JDD, SPD, and LSD only exhibited two biomarkers, which was the least (Figures 6C,D).

### 3.5. Functional prediction of the fecal microbiota

**Figure 7:**
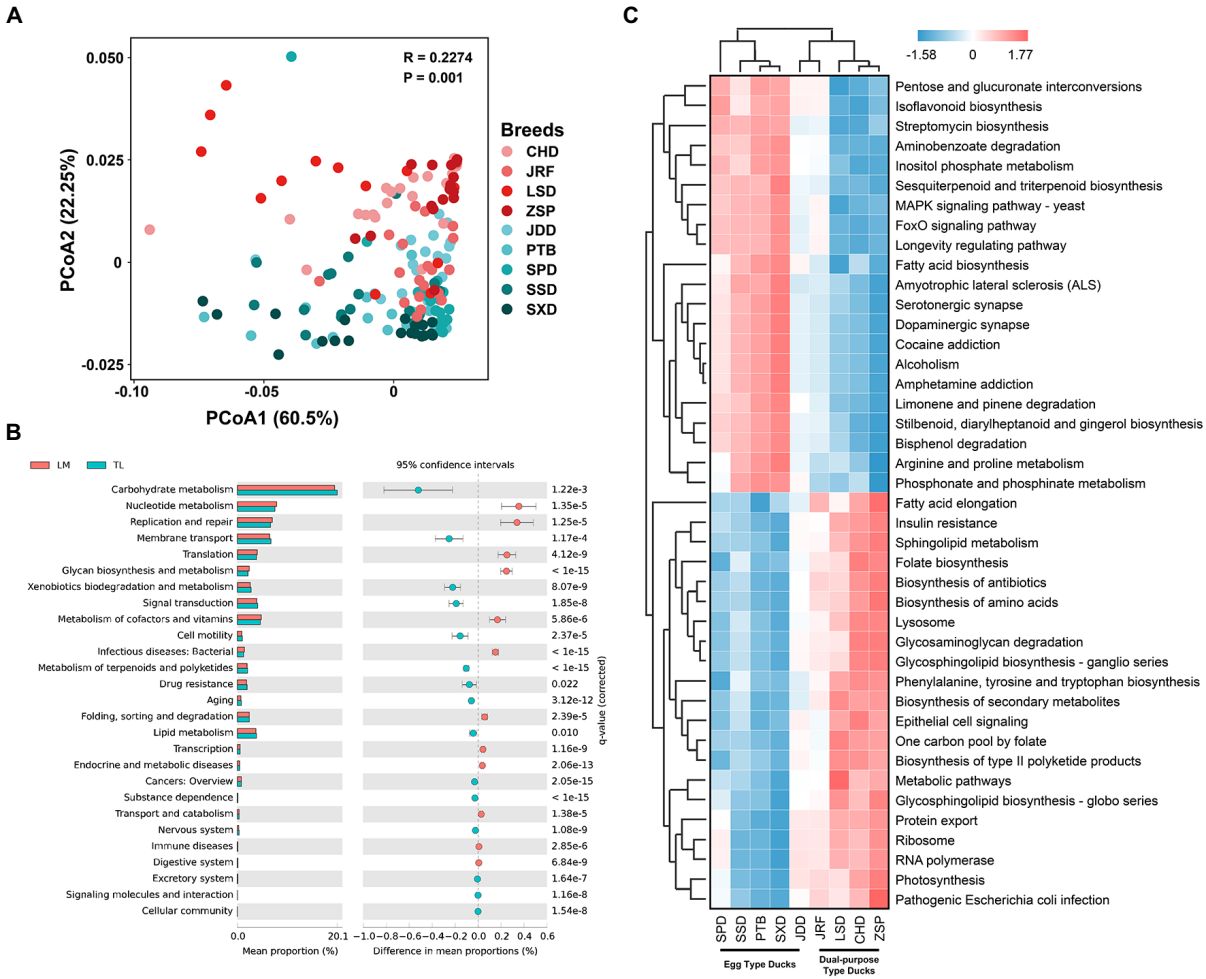
Fecal microbial functional prediction using Tax4Fun. **(A)** Principal coordinate analysis (PCoA) of fecal microbial functions. **(B)** Differences in fecal microbial function between the dual-purpose and egg type ducks based on Kyoto Encyclopedia of Genes and Genomes (KEGG) secondary metabolic pathways. **(C)** Heatmap of KEGG Orthology significantly enriched in fecal microbiota of dual-purpose and egg type ducks. Welch’s t-test and Storey’s methods were used for multiple tests adjustment.

The function of the gut microbiota in Chinese ducks was predicted using Tax4Fun. PCoA of the functional prediction showed that the functional characteristics of intestinal microbiota were separate for egg and dual-purpose type ducks (Figure 7A). There were 27 KEGG secondary metabolic pathways with significant differences between the two economic-use groups. The gut microbiota of the egg type ducks were mainly involved in carbohydrate metabolism, membrane transport, xenobiotic biodegradation and metabolism, signal transduction, metabolism of terpenoids, and polyketide and lipid metabolism. Additionally, transcription, translation, replication and repair, nucleotide metabolism, metabolism of cofactors and vitamins, and glycan biosynthesis and metabolism were enriched in the dual-purpose type ducks (Figure 7B). Moreover, a total of 43 KEGG Orthology (KO) pathways were identified which were significantly enriched in the GIT of Chinese ducks and cluster analysis was performed. In egg type ducks, the environmental information processing, carbohydrate metabolism, metabolism of terpenoids and polyketides, xenobiotic biodegradation and metabolism, and biosynthesis of other secondary metabolites had a higher abundance. Conversely, the genetic information processing, bacterial infectious diseases (including insulin resistance, pathogenic *Escherichia coli* infection, and *Helicobacter pylori* infection), biosynthesis of amino acids, antibiotics, secondary metabolites, glycan biosynthesis and metabolism, and fatty acid elongation were significantly enriched in the GIT of dual-purpose type ducks (Figure 7C).

## 4. Discussion

In recent years, with the increasing demand for poultry products, maintaining healthy and efficient growth of poultry has become more important. Studies have shown that the microbiota in different parts of the GIT exhibit different effects on the production performance and health of poultry (Pan and Yu, 2014; Diaz Carrasco et al., 2019). Moreover, the development of sequencing technologies has made it possible to explore the correlation between the gut microbiota and the performance and health of Chinese ducks.

### 4.1. Composition and differences of the fecal microbiota

In this study, 169 ducks divided into egg and dual-purpose type ducks from nine representative Chinese duck breeds were raised in the same growing environment. There was no significant difference in fecal microbial richness between egg and dual-purpose type ducks, but the fecal microbial alpha diversity in egg type ducks was significantly higher than that in dual-purpose type ducks. In production, the body size of dual-purpose type ducks are significantly larger than that of egg type ducks. This was consistent with the observation that the gut microbial diversity in obese individuals was significantly lower than that in normal individuals (Thingholm et al., 2019). At the phylum level, the fecal microbiota in the Chinese ducks were mainly composed of Firmicutes, Actinobacteriota, Bacteroidota, and Proteobacteria (≥ 80%). Similar to previous studies, Firmicutes were the most common dominant phylum (Zhao et al., 2018; Zhu et al., 2020). It has been widely recognized that the structure and diversity of the microbiota could be affected by different genetic backgrounds. The difference of the fecal microbial community had a significant effect on the different economic uses of the Chines ducks, including the change of the Firmicutes/Bacteroidetes ratio. The higher Firmicutes/Bacteroidetes ratio has been associated with human obesity (Turnbaugh et al., 2006; Zhang et al., 2009), whereas the lower ratio has been connected with weight loss (Ley et al., 2006). As the dominant beneficial microbial community in the human gut, both Firmicutes and Bacteriodetes have been linked with short chain fatty acid metabolism. More specifically, Firmicutes contributes to the synthesis of propionate and butyrate, whereas Bacteroidetes primarily synthesizes propionate. Compared with Firmicutes, Bacteriodetes can produce amylase and various glycosidases and break down starch and other polysaccharide substances (Polansky et al., 2015). Actinobacteriota, a phylum commonly found in the GIT of poultry, has an important role in the development and maintenance of intestinal homeostasis (Ocejo et al., 2019). Actinobacteriota occupies a high proportion in the feces of Chinese ducks. In this study, high abundance of Enterococcaceae in Chinese ducks is common, and the genus Streptococcus comprises pathogens, and opportunistic pathogens for humans and animals, some of which are associated with disease infection (Haenni et al., 2018). In addition, Lactobacillaceae is often considered as intestinal beneficial bacteria (Azad et al., 2018). *Clostridium sensu stricto 1*, *Bacteroides*, and *Alistipes* were relatively enriched in dual-purpose type ducks compared with egg type ducks. *C. sensu stricto 1* belongs to *Clostridium* spp., which can produce SCFAs such as acetic, propionic, and butyric acids. *Bacteroides* is a gram-negative anaerobic bacterium of Bacteroidaceae, which is usually associated with the synthesis of SCFAs (Saxena et al., 2016). SCFAs can regulate intestinal blood flow, stimulate the growth and proliferation of intestinal cells, regulate the production of mucin, and affect intestinal immune response (Pan and Yu, 2014), which might improve the disease resistance of dual-purpose type ducks. In contrast, the high abundance of *Erysipelatoclostridium* in egg type ducks may be connected to the reducing feed/egg ratio of egg type ducks (Guo et al., 2018), but it has also been identified as an opportunistic pathogen (Han et al., 2018), which may be linked to metabolic syndrome and gout (Shao et al., 2017). In conclusion, the change in the gut microbiota may affect the intestinal health and production performance of egg and dual-purpose type ducks.

The genetic resources and economic use were the main variables within the principle coordinates analysis; however, separation by the different breeds also uncovered distinct clustering manifesting a host component in microbiome composition, which is in agreement with previous studies (Zhao et al., 2013). The core fecal microbiome was represented by 39 genera, including *Bifidobacterium*, *Bacteroides*, *Lactobacillus*, *Clostridium*, *Anaerosporobacter*, *Faecalibacterium*, *Staphylococcus,* and *Bacillus*, all of which might promote the digestion of duck food in terms of hydrolyzing polysaccharides and other macromolecules, and the subsequent formation of SCFAs *via* fermentation which are then absorbed by the host (Pandit et al., 2018). Potential pathogenic and zoonotic organisms within the genera *Campylobacter*, *Erysipelatoclostridium*, *Staphylococcus*, *Fusobacterium,* and *Escherichia-Shigella* were also detected in the two groups, although clinical disease was not reported (Kaakoush et al., 2014). LEfSe analysis and IndVal index results identified some of these genera as biomarkers of the two groups. These results were in accordance with IndVal index. The genus *Lactobacillus* and *Streptococcus* were mainly representative biomarkers in dual-purpose type ducks. As for *Lactobacillus*, although they are generally considered beneficial, have been associated with growth inhibition in broilers due to competition in nutrient uptake or reduced fat uptake associated with bile acid binding(Knarreborg et al., 2002; Harrow et al., 2007). However, some studies showed that only the relative number of *Lactobacillus* in the cecum contents was positively correlated with the weight gain of broilers, while no significant statistical support for this correlation (Rubio et al., 2015). *Corynebacterium*, *Brevibacterium*, *Brachybacterium*, and *Romboutsia* were mainly representative biomarkers in egg type ducks. In humans, *Corynebacterium* is one of the main pathogens of bacterial pneumonia and has been reported as the pathogen of lower respiratory tract infection since the 1970s (Yatera and Mukae, 2020). *Romboutsia ilealis*, a member of the *Romboutsia* genus in *Peptostreptococcaceae*, is present in the human gut and is associated with the health status of polyps and colorectal cancer patients who lack the genus compared to healthy people, which may represent a microbial indicators of potential disease (Ricaboni et al., 2016; Mangifesta et al., 2018).

### 4.2. Functional prediction of the fecal microbiota

The metabolic function and pathways of gut microbiota in Chinese ducks were inferred using Tax4Fun. According to the functional prediction and statistical tests of the intestinal microbiota of ducks, a total of 27 secondary metabolic and 43 KO pathways enriched in the GIT of Chinese ducks were identified. Moreover, metabolism of terpenoids and polyketides, xenobiotic biodegradation and metabolism, and biosynthesis of other secondary metabolites exhibited a higher abundance in egg type ducks. This might be attributed to the abundance of Actinobacteriota in the intestinal tract of egg type ducks, which can degrade organic matter and produce a variety of natural drugs, enzymes, and bioactive metabolites (Ventura et al., 2007). Additionally, the genetic information processing, bacterial Infectious diseases, glycan biosynthesis and metabolism, and fatty acid elongation were extremely active in the GIT of dual-purpose type ducks. In a study investigating the linkages between the microbiota and feed efficiency in Xiayan chickens revealed that genetic information processing was associated with higher feed efficiency, whereas energy metabolism was associated with lower feed efficiency (Du et al., 2020). Insulin resistance has also been shown to be related to obesity and may be one of the key causes of obesity and blood glucose metabolism disorder (Gurung et al., 2020). Bacterial diseases might be strongly associated with *Esche-Shigella* and *Streptococcus*, which was significantly enriched in the GIT of dual-purpose type ducks. Additionally, glycan biosynthesis and metabolism and fatty acid elongation was enriched in the GIT of dual-purpose type ducks, which might promote glycogen accumulation and fat production. The results of the fecal microbiota function prediction using Tax4Fun showed that there were some differences in the gut microbial function between egg and dual-purpose type ducks. These differences in may play a specific role in the intestinal metabolism of ducks, and are closely related to intestinal health, dietary habits, and production performance of ducks.

## 5. Conclusion

In conclusion, the study showed that the differences in gut microbial composition, community structure, and function of Chinese ducks divided into egg and dual-purpose type ducks were significant. Dual-purpose type ducks with high-abundance Firmicutes and *Lactobacillus* exhibit a strong ability for nutrient uptake, decomposition, and storage. However, the higher microbial alpha diversity and more complex community structure are the characteristics of the intestinal microbiota in egg type ducks, and the functional advantage of energy metabolism may improve bioavailability. We inferred that the metabolic characteristics of the host may be affected by the unique microbiota and functioning found in the intestines of different domestic duck populations. This study supports the need for good intestinal health of domestic ducks and the discovery of potential probiotics, which has certain guiding significance and reference value.

## Data availability statement

The data that support the results and conclusions of this original study are publicly available. This datasets were deposited in the Genome Sequence Archive (GSA) database, accession number PRJCA012298.

## Ethics statement

The animal study was reviewed and approved by Jiangxi Science and Technology Normal University. Written informed consent was obtained from the owners for the participation of their animals in this study.

## Author contributions

XY, JO, and HC conceptualized and organized the research. HC provided funding support. YL performed analyses and wrote the draft manuscript. YW, HT, SZ, YX, LW, CW, KL, and YG collected samples and performed experiments. XY and HC revised the paper. All authors contributed to the article and approved the submitted version.

## Funding

This study was funded by National Natural Science Foundation of China (31960644).

## Conflict of interest

The authors declare that the research was conducted in the absence of any commercial or financial relationships that could be construed as a potential conflict of interest.

## Publisher’s note

All claims expressed in this article are solely those of the authors and do not necessarily represent those of their affiliated organizations, or those of the publisher, the editors and the reviewers. Any product that may be evaluated in this article, or claim that may be made by its manufacturer, is not guaranteed or endorsed by the publisher.
