# Supplementary material for: Microbial diversity and community composition of fecal microbiota in dual-purpose and egg type ducks
Source: Front Microbiol. 2023 Mar 31;14:1092100. doi: 10.3389/fmicb.2023.1092100 (PMC10102352; doi:10.3389/fmicb.2023.1092100)
Supplement: Supplementary file 1 [file Data_Sheet_1.docx]

Supplemenary Material

# Supplementary Figures

#
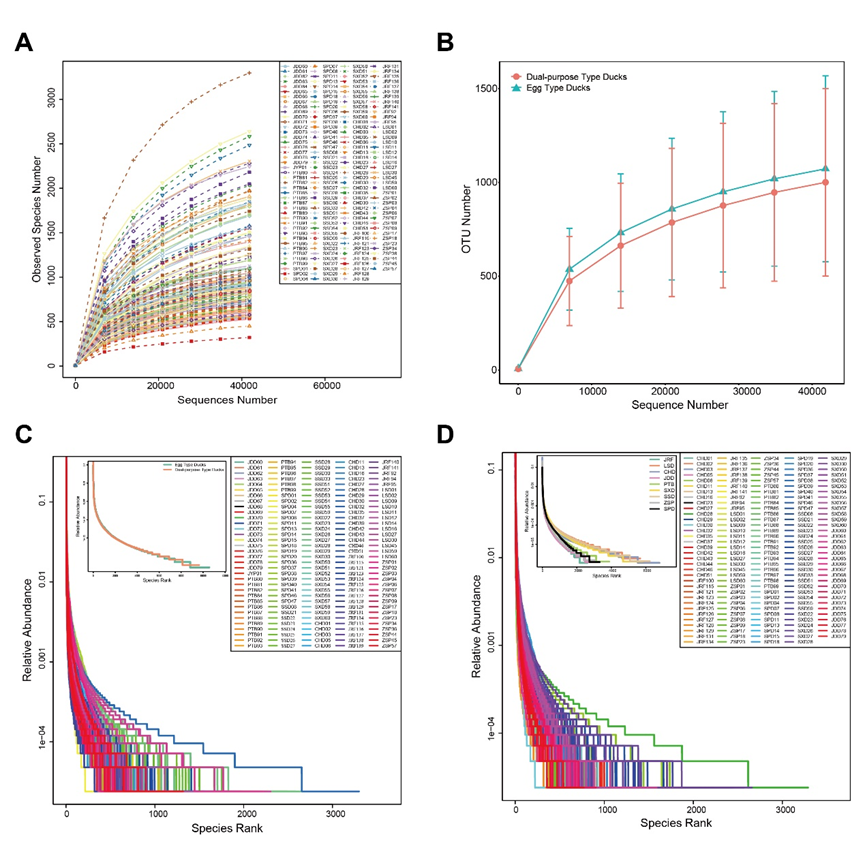


**Supplementary Figure 1.** **Species richness and diversity analysis of two groups and nine Chinese duck breeds.** Species rarefaction curves of each duck (**A**) and two groups (**B**). Rank-abundance curves of two groups (**C**) and nine Chinese duck breeds (**D**).


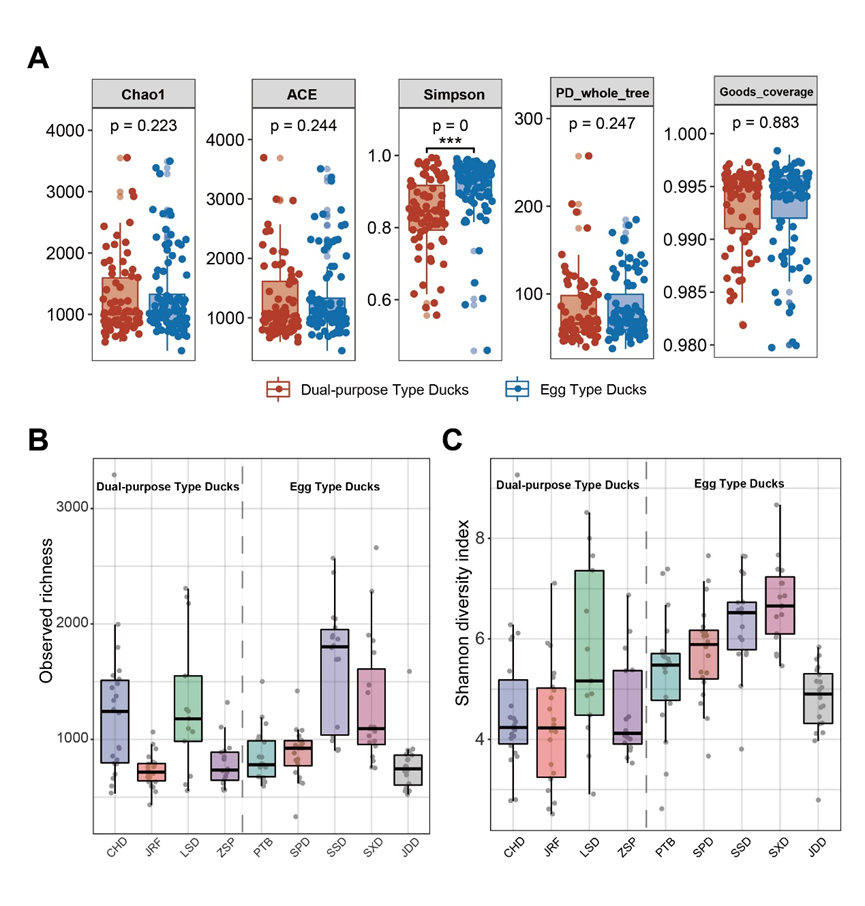


**Supplementary Figure 2. Comparison of fecal** **microbial Alpha diversity indices between two duck groups and nine Chinese duck breeds.** (**A**) Alpha diversity analysis of microbiota was compared according to the microbial diversity indices (Chao1, ACE, Simpson, PD whole tree, and Goods Coverage) between dual-purpose and egg type ducks. Observed richness (**B**) and Shannon (**C**) indices revealed the α diversity of nine Chinese indigenous duck breeds (∗ *P* < 0.05; ∗∗ *P* < 0.01; ∗∗∗ *P* < 0.001 for Kruskal-Wallis and Dunn's tests).


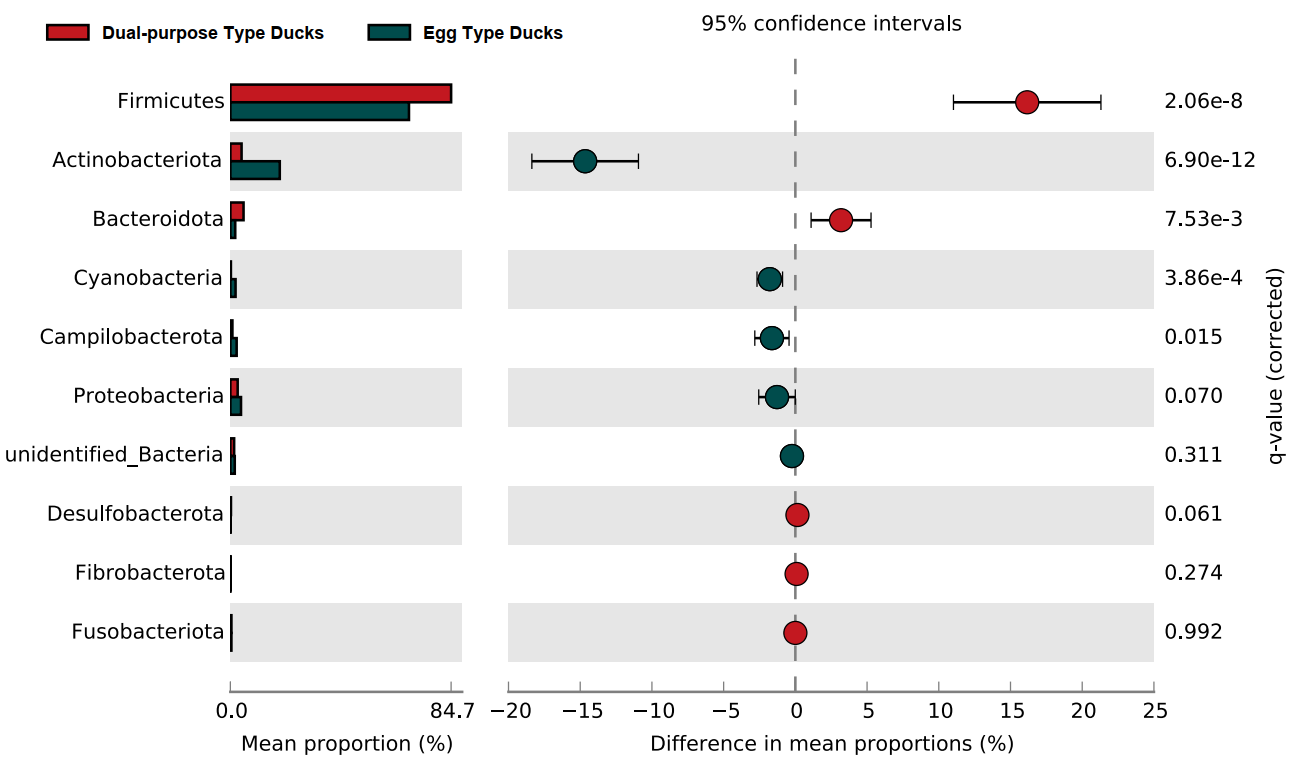


**Supplementary Figure 3. Differences significance analysis of the major microbial phylum.** The phylum with significant difference were determined using t-test (equal variance) by STAMP.


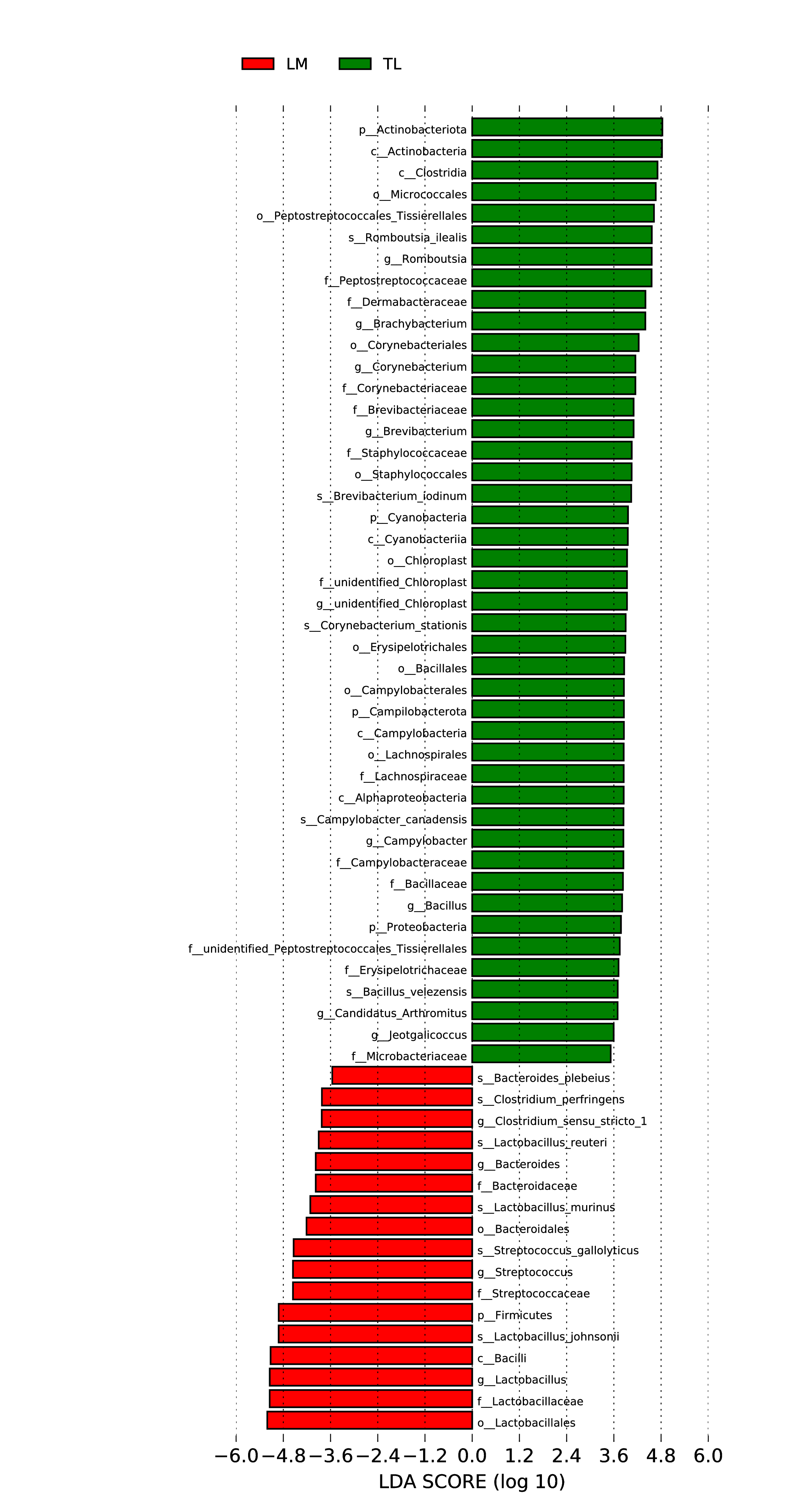


**Supplementary Figure 4. The biomarkers in Chinese ducks’ gut microbiota determined by LEfSe among two groups.** The gut microbiota of two groups were compared and determined as biomarkers using Kruskal-Wallis test (P < 0.05) with LDA score > 3.5.
